# Learning Gravity: An Innately Constrained Bayesian Model of Human Vestibular Development

**DOI:** 10.3390/audiolres16050135

**Published:** 2026-09-11

**Authors:** Giacinto Asprella Libonati, Fernanda Asprella Libonati

**Affiliations:** 1 UOC Otorhinolaryngology and Head and Neck Surgery, “Madonna delle Grazie” Hospital, ASM Basilicata, 75100 Matera, Italy; 2 Independent Researcher, 75100 Matera, Italy; fernandasprella@gmail.com

**Keywords:** vestibular development, gravity, Bayesian inference, internal models, predictive processing, developmental plasticity, fetal development, postural control, sensory reweighting, microgravity, aging, vestibular compensation

## Abstract

The vestibular apparatus is substantially organized before birth, whereas human equilibrium emerges only gradually through head control, sitting, standing, and independent walking. We propose an Innately Constrained Bayesian Model of Vestibular Development in which a phylogenetically shaped architecture constrains an initial model space; prenatal and postnatal experience calibrate internal models of self-motion and gravity; and developmentally gated plasticity modulates the rate of updating. We explicitly distinguish established evidence from interpretations and model-derived hypotheses. Recent human and nonhuman primate work on internal models, active self-motion, and multisensory recalibration is integrated with developmental and clinical evidence. The framework is then tested conceptually across congenital and acquired vestibular loss, altered gravity, and aging. Its proposed novelty lies not in Bayesian inference or internal models themselves, but in combining phylogenetic constraints, prenatal calibration, a qualitative transition at birth, time-dependent plasticity, and life-span context switching within a single developmental account. Finally, we operationalize the model through measurable predictions involving VOR adaptation, sensory-weighting coefficients, anticipatory postural responses, context-specific readaptation, and postural stability. The framework is intended as a falsifiable perspective rather than a definitive mechanistic account.

## 1. Introduction and Aim

Human balance is conventionally described as the integration of vestibular, visual, proprioceptive, and somatosensory information. This description identifies the contributing signals, but it does not fully explain how the developing nervous system learns their meaning, estimates their reliability, and transforms them into a coherent representation of the body moving within Earth’s gravitational field. The peripheral vestibular apparatus is substantially organized before birth, whereas head control, sitting, standing, and independent walking emerge over a prolonged postnatal period. Mature equilibrium therefore cannot be understood as the simple activation of a fully formed controller. Rather, it requires the progressive construction, testing, and recalibration of internal models that link sensory evidence, motor commands, body mechanics, and environmental regularities [1,2,3,4].

The aim of this Perspective is to propose an Innately Constrained Bayesian Model of Vestibular Development that integrates three levels that are usually considered separately: phylogenetically shaped vestibular architecture, experience-dependent ontogenetic calibration, and context-sensitive updating across the life-span. The term Bayesian is used at the computational level to describe the combination of prior expectations with uncertain sensory evidence according to their relative precision; it does not imply that the nervous system performs explicit symbolic probability calculations [2,3,5]. We further distinguish throughout the paper among established evidence, interpretation of existing findings, and hypotheses generated by the proposed model.

## 2. Evolutionary Constraints and Three-Dimensional Vestibular Architecture

Ontogenetic learning occurs within a phylogenetically constrained model space. The vertebrate vestibular system evolved under the persistent constraint of terrestrial gravity, while habitual bipedalism in the hominin lineage increased the mechanical demands of an upright stance, gaze stabilization, and anticipatory postural control. Natural selection did not provide a complete repertoire of postural solutions; it provided receptors, pathways, reference frames, and sensorimotor couplings that make some interpretations of motion and gravity biologically available. In this limited sense, the inherited organization can be considered a species-level generative architecture: a constrained set of possible solutions from which individual postural behavior can be learned [1,2,6].

The labyrinth itself provides a preconfigured three-dimensional sampling system. The utricular and saccular maculae offer complementary, distributed representations of gravitoinertial acceleration; because their sensory epithelia are curved and contain hair-cell populations with different polarization vectors, they should not be reduced to two Cartesian detectors. The three semicircular canals are arranged in approximately orthogonal planes and encode angular head motion broadly corresponding to yaw, pitch, and roll. The lateral canal is not exactly earth-horizontal in the anatomical upright position and is commonly described as inclined upward anteriorly by approximately 20–30 degrees, so a modest forward head tilt brings it closer to the horizontal plane [7]. Bilateral push–pull organization, vestibulo-ocular and vestibulospinal pathways, and cerebellar computations provide a structured basis for estimating self-motion and orientation. Importantly, the otolith signal is intrinsically ambiguous because it reflects gravitoinertial force, and cannot, by itself, distinguish tilt from translation; central inference is therefore a fundamental feature of vestibular processing [2,3,4].

The analogy with a generative grammar is therefore heuristic rather than literal. The infant does not inherit the ‘sentences’ of postural behavior, but the biological conditions that make a structured postural repertoire learnable. Phylogeny constrains what the system is prepared to learn; ontogeny determines how accurately, efficiently, and flexibly that architecture is calibrated to the dimensions and capabilities of the individual body.

## 3. Prenatal Calibration, Birth, and Developmentally Gated Plasticity

The fetus is continuously exposed to Earth’s gravity, but its biomechanical consequences are modified by amniotic buoyancy, uterine containment, maternal support, flexed posture, and the absence of independent weight-bearing. Prenatal life is therefore not a state of microgravity; it is gravity experienced through a supported, contained, and partially unloaded body. Vestibular stimulation is prominent and arises from maternal walking and turning, respiratory and postural movements, passive displacement, and spontaneous fetal head and body movement. Fetal experience is not exclusively vestibular: tactile, proprioceptive, somatic, auditory, and vibratory information are also present. What is restricted is the postnatal configuration of multisensory experience, including structured vision, plantar support, autonomous axial loading, extended optic flow, stable external landmarks, and mature action–consequence contingencies [1,8,9].

Interpretation: These observations make it plausible that prenatal experience contributes to preliminary priors concerning motion continuity, periodicity, acceleration, orientation, and vestibular–somatic concurrence. Direct evidence for human fetal vestibular learning, however, remains limited, and this proposal should be regarded as an inference from developmental anatomy, fetal behavior, neonatal observations, and animal work rather than as established human physiology.

Birth is not the first encounter with gravity; it is a qualitative transition from supported gravitational exposure to independent biomechanical interaction with gravity. Buoyant support is lost, stable support surfaces become behaviorally relevant, structured visual input expands, and self-generated head and body movements produce new combinations of canal, otolith, optic-flow, cervical, cutaneous, and motor signals. Prenatal expectations may remain partially useful, but they cannot fully predict these new multisensory consequences. Birth therefore initiates a prolonged period of model mismatch and recalibration rather than a single discrete prediction error.

Developmental plasticity provides a plausible physiological substrate for this recalibration. In developing rats, Grassi, Dieni, Frondaroli, and Pettorossi showed that visual experience influences a developmental shift in glutamatergic synaptic plasticity within the medial vestibular nucleus from a predominance of long-term depression (LTD) toward long-term potentiation (LTP) during the period in which visual input becomes available to contribute to vestibulo-ocular reflex (VOR) adaptation [10,11,12]. This is an important mechanistic proof-of-principle for experience-dependent developmental gating, but it must not be treated as direct evidence of an identical synaptic transition or timetable in humans. No in vivo human demonstration of this LTD-to-LTP developmental shift is currently available.

Model hypothesis: We therefore represent developmental updating conceptually as M(t + 1) = M(t) + α(t) × Π(t) × ε(t) × E(t), where α(t) is a development-dependent learning rate or plastic potential, Π(t) is the precision assigned to sensory evidence, ε(t) is the prediction error, and E(t) represents the quality and recurrence of experience. The equation is not intended as a validated quantitative law. Its purpose is to make explicit the hypothesis that the effect of experience depends not only on stimulus magnitude or repetition, but also on when the experience occurs relative to circuit maturation.

## 4. Bayesian Updating and Active Postnatal Learning

The developing nervous system must continuously estimate body and environmental state from incomplete and sometimes conflicting evidence. Bottom-up signals convey acceleration, orientation, optic flow, muscle state, pressure, and support; top-down processes generate expectations about body position, gravitational direction, environmental stability, and the likely consequences of action. In a simplified form, the posterior estimate P(X|S,A) is proportional to the likelihood P(S|X,A) multiplied by the prior P(X), where X is the inferred body–environment state, S is multisensory evidence, and A is action or intention [2,3,5].

The predictive character of vestibular processing is supported by work in humans and nonhuman primates. Recent reviews emphasize that vestibular cerebellar circuits combine vestibular and extra-vestibular information to build internal models of eye, head, and body motion and their orientation relative to gravity [4]. In rhesus macaques, Purkinje cells in the anterior vermis combine sensory and motor-related signals to predict the sensory consequences of active self-motion, providing a mechanistic basis for suppressing expected vestibular reafference [13]. Population recordings in macaque early vestibular pathways further show coding adapted to the statistics of natural self-motion [14]. These findings do not establish the proposed developmental model, but they provide strong primate evidence that predictive and context-sensitive vestibular computations exist in the mature system.

Active and passive motion are therefore not equivalent learning conditions. Self-generated movement permits comparisons to be made between a motor-based prediction and its actual sensory consequences, whereas passive motion lacks the same efference-copy structure. Human work on agency and self-motion likewise supports a role for predictive mechanisms in weighting visual and vestibular information [15], and recent work on cross-modal plasticity emphasizes that visual–vestibular recalibration remains dynamic throughout life [16].

As motor abilities expand, each developmental milestone creates a new learning environment. Head control, rolling, sitting, standing, and walking introduce progressively different combinations of gravitational torque, support-surface information, optic flow, self-generated acceleration, and postural error. The child therefore does not simply acquire a sequence of reflexes; the child learns increasingly transferable relationships between action and consequence. This process can be viewed as a progression from predominantly reactive correction toward increasingly anticipatory control [6,17,18,19,20,21].

## 5. What Is New in the Present Model?

Bayesian inference, internal models, sensory reweighting, predictive coding, and vestibular adaptation are not new concepts, and the present Perspective does not claim otherwise. Bayesian approaches have long been used to explain gravitoinertial ambiguity and multisensory integration [3,5], while internal-model accounts have been developed for cerebellar and vestibular computations [2,4,22]. Recent work has further clarified predictive processing during active self-motion and cross-modal recalibration [13,14,15,16].

The proposed contribution is instead based on the integration of these established concepts into a developmental and life-span framework with four specific features. First, it treats phylogenetically shaped vestibular geometry and circuitry as constraints on an initial model space rather than as a preformed balance controller. Second, it distinguishes prenatal calibration from the qualitative multisensory and biomechanical transition at birth. Third, it introduces developmentally gated plasticity, α(t), as a factor that modulates how strongly prediction error can update the model at different developmental stages. Fourth, it distinguishes four computational situations that are often conflated: first model construction during development, lesion-driven compensation after vestibular loss, environment-driven recalibration in altered gravity, and adaptation to declining sensory precision during aging.

This synthesis is intended to generate experimentally discriminable predictions rather than to rename established mechanisms. Its value therefore depends on whether it yields measurable differences in VOR adaptation, sensory weighting, anticipatory postural control, context switching, and rehabilitation outcome that are not captured as clearly by a purely reflex-based or static sensory-integration account.

## 6. Life-Span Tests of the Model: Vestibular Loss, Altered Gravity, and Aging

Congenital or early-onset vestibular dysfunction provides a test of model construction under degraded sensory evidence. A child with severe bilateral vestibular loss must construct balance from the outset using alternative signal weighting, whereas an individual who acquires vestibular loss after stable head control and locomotion can recalibrate previously established representations. Recent pediatric data support the clinical relevance of this distinction: vestibular impairment is associated with delayed postural milestones and reduced motor competence, and the current pediatric consensus recommends vestibular assessment in children with imbalance, gross-motor delay, or relevant hearing loss [18,20,21,23,24]. The model predicts that congenital and later-acquired losses should differ not only in reflex metrics, but also in anticipatory control, visual dependence, and generalization across contexts.

Altered gravity provides a complementary adult test in which the sensory apparatus is relatively intact while environmental contingencies change. During orbital spaceflight, the usual terrestrial relationship among otolith activity, body weight, plantar loading, proprioception, and gravitational vertical is altered. Postflight disturbances of stance, locomotion, gaze stabilization, and orientation are consistent with context-specific recalibration and subsequent readaptation to 1 g [25,26,27]. The model therefore treats microgravity as environment-driven recalibration of an established system, not as an analog of fetal life or congenital vestibular deficiency. Hypergravity and high-performance aviation offer related but more complex paradigms because the imposed gravitoinertial forces are direction-dependent and interact with visual, instrumental, cervical, and training-related factors [15].

Aging presents the opposite challenge: the gravitational environment remains stable while sensory precision and motor efficiency decline. Age-related vestibular loss, visual and somatosensory changes, slower integration, and reduced motor reserve increase uncertainty and can drive compensatory reweighting [28,29,30,31,32]. We retain the hypothesis of an adaptive vestibular posture, but define it operationally as a reproducible head–trunk configuration associated with measurable improvement in postural stability or reduction in sensory conflict relative to alternative configurations, after accounting for musculoskeletal constraints. The hypothesis remains unproven. Testing it requires three-dimensional head and trunk kinematics, imaging-derived canal orientation, vestibular testing, cervical proprioceptive assessment, posturography, and gait analysis [7,28,29,30,31]. These distinct life-span conditions and their predicted adaptive profiles are summarized in Table 1.

## 7. Testable Predictions and Clinical Implications

A theoretical model is useful only if its constructs can be operationalized. Table 2 therefore links each major prediction to an experimental paradigm and a quantifiable read-out. The central predictions are that active self-generated motion should produce greater retention or transfer than matched passive stimulation; major motor milestones should be accompanied by measurable changes in sensory weighting and anticipatory control; congenital and acquired vestibular loss should differ in prediction-related metrics even when peripheral deficits are comparable; altered-gravity transitions should reveal context-specific adaptation and readaptation time constants; and age-related postural configurations should be considered adaptive only if they confer a measurable stability benefit.

The same framework has clinical implications. Vestibular dysfunction should not be defined solely by receptor or reflex deficit, because the functional phenotype also depends on the pre-existing model, timing of the lesion, precision of alternative signals, plasticity, and motor capacity. Vestibular phenotype can therefore be represented conceptually as f(signal precision, prior model, plasticity, context, motor capacity). Compensation may involve the construction of a new probabilistic solution in which residual vestibular information, vision, proprioception, somatosensation, and motor strategies are assigned new weights according to their reliability [4,11,12,33].

Rehabilitation can similarly be reframed as the deliberate provision of structured and behaviorally meaningful prediction errors. This perspective favors active movement, variable contexts, manipulation of sensory reliability, and assessment of transfer to untrained tasks rather than repetition of a single stereotyped exercise. In children, the developmental timing of intervention may be especially important if learning rate is indeed gated by sensitive windows; in older adults, the limiting factor may instead be reduced precision and slower updating. These implications remain hypotheses to be tested prospectively.

## 8. Limitations and Conclusions

The model has important limitations. Direct evidence for human fetal vestibular learning is sparse. The proposed developmental plasticity window is supported by rodent physiology, not by direct in vivo human evidence, and no assumption should be made that the human system follows the same synaptic timetable. The recent nonhuman primate literature strongly supports predictive vestibular computations during active self-motion [13,14], but these studies examine mature animals and do not demonstrate the developmental trajectory proposed here. Bayesian inference, predictive processing, internal models, and active inference are related but non-identical frameworks; the present model uses them at a computational level without specifying a unique cellular implementation.

Motor development is also influenced by vision, proprioception, cerebellar function, muscle strength, cognition, motivation, caregiving, and opportunities for movement. The model must therefore be compared with dynamic-system, ecological, embodied, and conventional sensory-integration accounts. Its scientific value will depend on whether it produces more precise, falsifiable predictions and improves assessment or rehabilitation.

**Figure 1:**
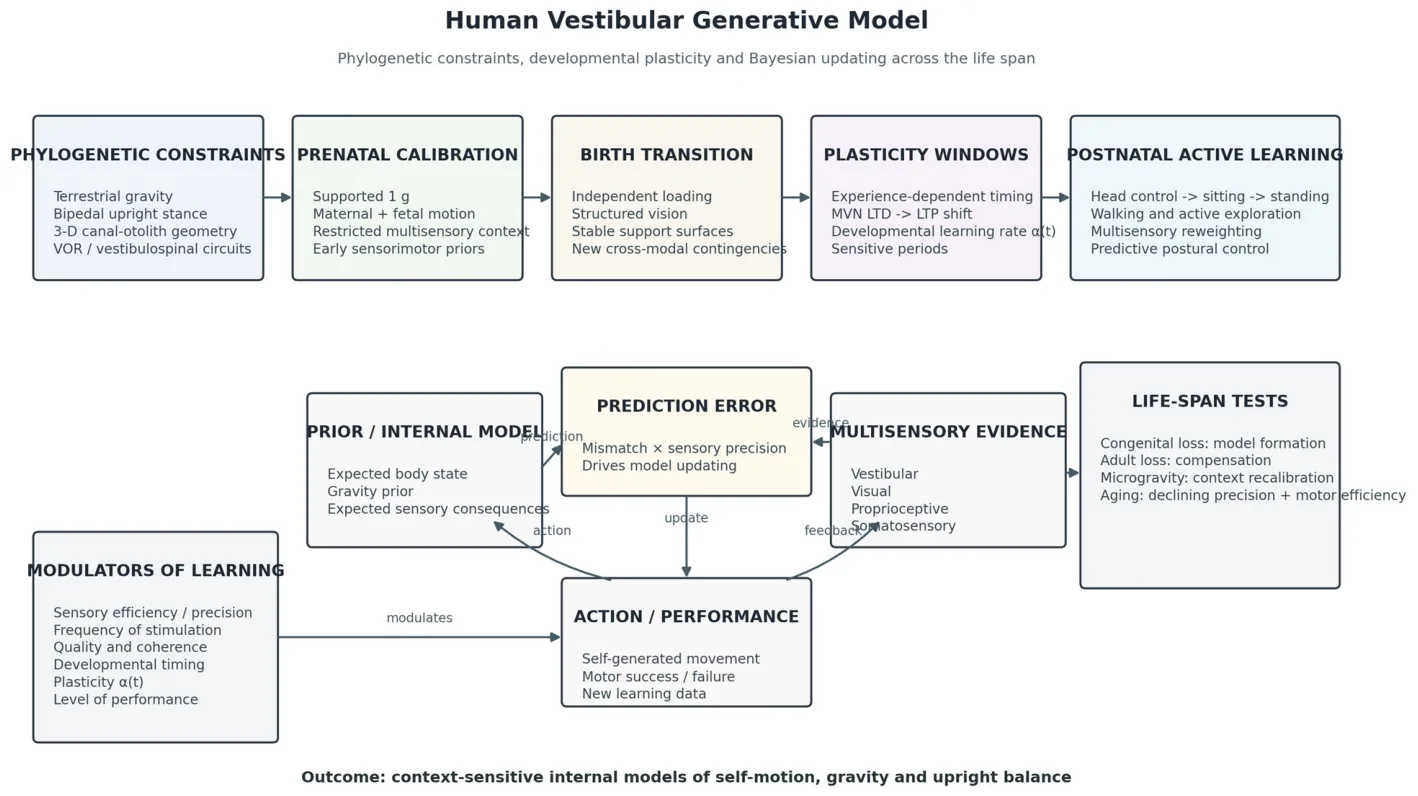
Human vestibular generative model. Phylogenetic constraints, prenatal calibration, the transition at birth, developmental plasticity, and postnatal active learning are integrated with a recurrent prediction-error loop. The figure separates life-span challenges—congenital loss, acquired loss, altered gravity, and aging—from the developmental sequence. The LTD-to-LTP shift in the rat medial vestibular nucleus is shown as an experimental proof-of-principle for experience-dependent developmental plasticity, not as established human physiology.

The central proposition is that the human vestibular system is neither a blank slate nor a complete equilibrium controller at birth. Evolution provides a structured three-dimensional architecture; prenatal experience may begin its calibration; birth transforms the causal and multisensory environment; developmentally gated plasticity modulates learning; and active postnatal behavior progressively constructs predictive models of the body in gravity. Across the life-span, congenital loss, acquired lesions, altered gravity, and aging can be viewed as distinct challenges to different components of the same adaptive system. The overall architecture of the proposed framework is summarized in Figure 1.

## Figures and Tables

**Table 1:** Development, altered gravity, vestibular loss, and aging as distinct tests of the vestibular generative model.

Condition	State of Vestibular System	Environment	Primary Computational Problem	Expected Adaptation	Plasticity Condition
Early development	Maturing, incompletely calibrated	Terrestrial 1 g; rapidly expanding multisensory context	First construction of body-in-gravity models	Acquisition of priors, sensory weights, predictive postural control	High and time-dependent; sensitive windows
Congenital/early vestibular loss	Absent, reduced, or unreliable input during development	Terrestrial 1 g	Model construction without reliable vestibular evidence	Atypical weighting/model formation; delayed milestones	Strongly dependent on timing relative to plasticity windows
Adult acquired vestibular loss	Previously calibrated system with new peripheral deficit	Terrestrial 1 g	Recalibration after unexpected signal degradation	Compensation, substitution, contextual reweighting	Compensatory plasticity acting on established models
Microgravity	Substantially efficient mature system	Altered gravitational contingencies/near-weightlessness	Recalibration of an established terrestrial model	Context-specific reweighting and readaptation to 1 g	Adaptive plasticity in a mature system
Hypergravity/high acceleration	Efficient trained system exposed to extreme gravitoinertial loads	Transient, direction-dependent high-g conditions	Interpretation of ambiguous canal-otolith and visual–instrumental evidence	Training-dependent contextual expectations and strategies	Experience- and training-dependent
Senescence	Declining sensory precision and motor efficiency	Stable terrestrial 1 g	Preservation of balance with noisy inputs and reduced response capacity	Compensatory reweighting, behavioral restriction, possible adaptive posture	Reduced/slower, with individual reserve

**Table 2:** Operational definitions and quantifiable predictions generated by the model.

Model Prediction	Experimental Paradigm	Quantifiable Read-Out	Expected Direction
Active > passive learning	Matched active and passive head-motion training in infants/children or adults	Change in VOR gain; retention at 24–48 h; transfer to untrained frequencies/tasks	Greater retention and transfer after active self-generated movement
Milestones alter sensory weighting	Longitudinal assessment before/after head control, sitting, standing, walking	Vestibular/visual/somatosensory weighting coefficients; sway gain; anticipatory EMG latency	Stage-specific reweighting and shorter anticipatory latencies with new motor competence
Congenital ≠ acquired loss	Matched cohorts with comparable bilateral peripheral deficits	Visual dependence; sensory-weighting coefficients; anticipatory postural adjustments; transfer across contexts	Greater atypical weighting and impaired anticipatory generalization in congenital loss
Plasticity is developmentally gated	Age-stratified VOR adaptation or rehabilitation paradigms	Learning-rate parameter α; VOR adaptation time constant; retention	Age-/stage-dependent learning rates rather than uniform updating
Context-specific gravity models	Pre/post spaceflight or centrifuge exposure	Readaptation time constant; VOR/postural aftereffects; sensory weighting	Distinct adaptation/readaptation profiles consistent with context selection
Adaptive vestibular posture	3D head/trunk kinematics + CT-derived canal orientation + posturography	Canal-to-earth angle; CoP sway; gait variability; sensory-conflict cost	A posture qualifies as adaptive only if stability improves after covariate adjustment
Active multisensory rehabilitation improves generalization	Randomized active-variable vs. passive/stereotyped rehabilitation	Untrained-task performance; dynamic visual acuity; postural transfer; patient-reported function	Greater generalization in active, variable, context-rich training

## Data Availability

No new data were created or analyzed in this study. Data sharing is not applicable to this article.
